# Association between Gait Deviation Index and Physical Function in Children with Bilateral Spastic Cerebral Palsy: A Cross-Sectional Study

**DOI:** 10.3390/jcm9010028

**Published:** 2019-12-20

**Authors:** Tadashi Ito, Koji Noritake, Hiroshi Sugiura, Yasunari Kamiya, Hidehito Tomita, Yuji Ito, Hideshi Sugiura, Nobuhiko Ochi, Yuji Yoshihashi

**Affiliations:** 1 Three-Dimensional Motion Analysis Room, Aichi Prefectural Mikawa Aoitori Medical and Rehabilitation Center for Developmental Disabilities, Okazaki 444-0002, Japan; 2 Department of Physical Therapy, Graduate School of Medicine, Nagoya University, Nagoya 461-8673, Japan; hsugiura@met.nagoya-u.ac.jp; 3 Department of Orthopedic Surgery, Aichi Prefectural Mikawa Aoitori Medical and Rehabilitation Center for Developmental Disabilities, Okazaki 444-0002, Japan; noritake@mikawa-aoitori.jp (K.N.); cedar164@ybb.ne.jp (H.S.); 4 Department of Orthopedic Surgery, Nagoya University Hospital, Nagoya 466-8560, Japan; yasunari.kamiya@gmail.com; 5 Graduate School of Health Sciences, Toyohashi Sozo University, Toyohashi 440-8511, Japan; h_tomita@sozo.ac.jp; 6 Department of Rehabilitation, Aichi Prefectural Mikawa Aoitori Medical and Rehabilitation Center for Developmental Disabilities, Okazaki 444-0002, Japan; yosihasi@mikawa-aoitori.jp; 7 Department of Pediatrics, Aichi Prefectural Mikawa Aoitori Medical and Rehabilitation Center for Developmental Disabilities, Okazaki 444-0002, Japan; yuji.ito@med.nagoya-u.ac.jp (Y.I.); aoi2pochi@yahoo.co.jp (N.O.)

**Keywords:** three-dimensional gait analysis, five-times-sit-to-stand test, Gait Deviation Index, gait speed, children with spastic cerebral palsy

## Abstract

This study examined the association between Gait Deviation Index (GDI) and the five-times-sit-to-stand test (FTSST) or gait speed results, which represent mobility and muscle strength of the lower extremities in ambulatory children with Gross Motor Function Classification System (GMFCS) level I and II spastic cerebral palsy. In this cross-sectional, observational study, three-dimensional gait analysis data were obtained during gait trials to evaluate the GDI in 35 children (age 5–16 years) with spastic palsy. Motor function was evaluated using FTSST and gait speed. Gross motor function was evaluated using GMFCS. Children with GMFCS level II spastic cerebral palsy demonstrated lower GDI (*p* < 0.001) and poorer FTSST (*p* = 0.031) than those with GMFCS level I spastic cerebral palsy. Correlation analysis showed that FTSST results were significantly correlated with GDI (*r* = −0.624; *p* < 0.001). Motor function may be important for the maintenance of gait quality in patients with GMFCS level I and II spastic cerebral palsy and should not be ignored. In conclusion, reduction in gait impairment may affect the values of FTSST and GDI in patients with spastic cerebral palsy who can ambulate without an assistive device.

## 1. Introduction

Gait Deviation Index (GDI) is an important tool that represents the overall gait pathology in patients with spastic cerebral palsy and other pathologies using numerical values and gait ability [1]. GDI is determined based on a score derived from the data points of the kinematics of three-dimensional gait analysis of the pelvis and hip in the sagittal, frontal, and horizontal planes; knee and ankle in the sagittal plane; and foot progression [1]. A ten-point reduction in GDI corresponds to one standard deviation from the mean value in healthy controls based on which the extent of gait pathology can be determined [1,2]. It has been demonstrated that GDI deteriorates with increasing severity of spastic cerebral palsy [1]. Several studies have demonstrated that reduced GDI correlates with gross motor function, as assessed using the Gross Motor Function Classification System (GMFCS) [3,4,5,6]. Sagawa et al., demonstrated that there is an important association between clinical parameters (range of motion, muscle strength, and spasticity) and GDI in participants with spastic cerebral palsy [7]. Although this finding suggests that gait deviations in participants with spastic cerebral palsy correlate with their impairments, clinicians often find it difficult to confirm this association despite the various available tests. There are several screening tests that represent a patient’s functional ability. For example, gait speed is a reliable tool for measuring the gait capacity in participants with spastic cerebral palsy, whereas the five-times-sit-to-stand test (FTSST) measures the functional muscle strength in the lower extremities and balance [8,9,10]. Furthermore, FTSST may indirectly reflect the gait status in patients with spastic cerebral palsy because its results are associated with functional muscle strength. Therefore, FTSST may be considered as an assessment of physical function, which in turn captures the overall impact of gait quality and is an important indicator of gait ability. In contrast, assessment of physical function using gait speed reflects that gait ability may be a valuable measure of gait quality in patients with spastic cerebral palsy. However, to our knowledge, there are no studies that have examined the association between FTSST or gait speed and GDI in patients with spastic cerebral palsy. Poor gait quality in patients with spastic cerebral palsy can lead to impaired mobility and disability, which can subsequently result in muscle weakness or a decline in balance. Therefore, gait quality assessment may be important in these patients. Assessment of gait quality could additionally help predict physical function, which could then be used in the prevention of physical function impairments. However, the specific physical function assessments that explain the relationship between GDI and spastic cerebral palsy remain unclear. The aim of this study was to investigate the association between GDI and FTSST or gait speed in patients with spastic cerebral palsy. We hypothesized that FTSST or gait speed could be suitably used in conjunction with the assessment of gait quality in these patients. 

## 2. Materials and Methods

### 2.1. Study Population

This cross-sectional study was performed between April 2016 and April 2019 in tandem with general clinical practice. The power analysis for sample size revealed that the optimal sample size for this study was 26 participants. We included 35 pediatric patients (age 5–16 years) with spastic cerebral palsy who underwent ambulatory treatment under the supervision of three pediatric orthopedic surgeons. We chose to exclude children below the age of 5 years because they do not have a stable walking pattern. The inclusion criteria were diagnosis of bilateral cerebral palsy, GMFCS levels I and II, and the ability to walk independently without assistive devices and to undergo clinical evaluations of FTSST and gait speed. The exclusion criteria were lower extremity orthopedic surgery within the past 1 year, botulinum toxin A injections within the past 6 months, cardiovascular disease, uncontrolled epilepsy, inability to communicate due to severe intellectual impairment, inability to walk without an assistive device, a Modified Ashworth Scale score of >2, intrathecal baclofen administration, athetoid or mixed types of cerebral palsy, ophthalmologic abnormalities (e.g., reduced visual acuity, strabismus) which impair gait function, and inability to complete physical and three-dimensional gait analysis tests. 

The legal guardians of all participants provided written informed consent for the study and for the publication of identifying information and images. The study was conducted in accordance with the Declaration of Helsinki, and the protocol was approved by the Ethics Committee of the Aichi Prefectural Mikawa Aoitori Ethics Review Board (approval number: 29002). The study was conducted in accordance with the Health Insurance Portability and Accountability Act of 1996 (HIPAA) Privacy Rule, and the manuscript was prepared in accordance with the STROBE guidelines.

### 2.2. Data Collection

**Figure 1:**
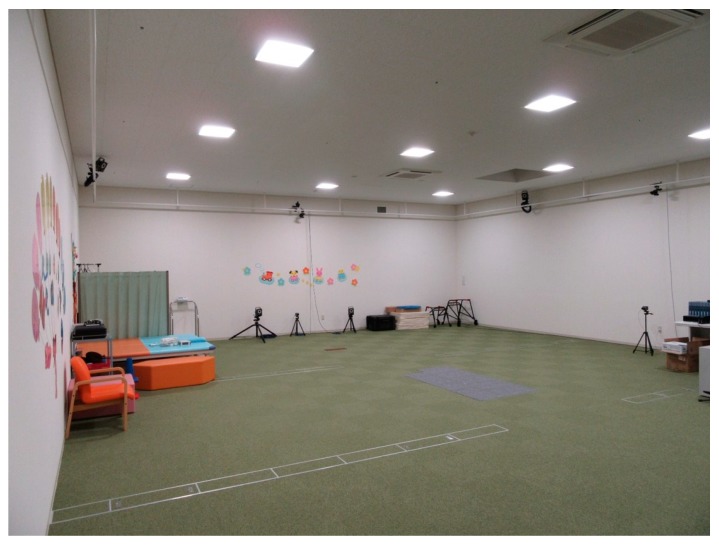
Three-dimensional motion analysis system. The system consists of eight strobe cameras mounted from the ceiling or floor and eight force plates placed in the middle of the walkway.

Instrumented three-dimensional gait analysis was performed using the Plug-In-Gait model to obtain lower extremity kinematics at the pelvis, hip, knee, and ankle of both sides. Measurements were performed using an eight-camera motion analysis system at a sampling frequency of 100 Hz (type MX-T 20S; Vicon, Oxford, UK) (Figure 1). After the collection of anthropometric data, participants were equipped with 16 retro-reflective markers (combination of the Plug-In-Gait lower body Ai; Vicon, Oxford, UK) by physiotherapists experienced in clinical gait analysis. The Plug-In-Gait lower body Ai marker set was placed on the anterior and posterior superior iliac spines, lateral femur, lateral knee joint, lateral lower limb, lateral malleolus of the ankle joint, head of the second metatarsal, and calcaneus on both sides. Following a static measurement trial in an upright position, the participants walked barefoot at their self-selected speed on 8-AMTI OPT force plates (Advanced Mechanical Technology, Inc., Watertown, MA, USA) until at least three trials were performed and subsequently recorded [5,11]. The data from three trials for each patient were exported from Polygon (Vicon Polygon 4.4; Vicon, Oxford, UK) to Excel.

### 2.3. GDI Model and GMFCS

The GDI of the participants was calculated as described by Schwartz and Rozumalski using the current pipeline of Vicon [1]. The mean GDI was calculated over three trials of gait analysis for the right and left lower limbs. GDI has been demonstrated to have high validity with excellent inter-trial and intra-rater reliability [3,12,13,14]. The GMFCS level was assessed and recorded by a trained pediatric orthopedic surgeon. 

### 2.4. Clinical Motor Function Evaluation

#### 2.4.1. FTSST

Initially, the participants sat on a chair with their arms crossed over the chest. Subsequently, they were asked to stand up and sit down five times as quickly as possible without using arm support [9,10]. The test began with the word “go” and stopped when the participants stood up after the fifth repetition, and the time duration was recorded using a stopwatch (seconds). FTSST results were determined using a non-dimensionalized FTSST equation:

(1) $$t\;*=t/\sqrt{gL_{leg}},$$

Where *t* is the absolute FTSST value, *L_leg_* is the length of the leg, and *g* is gravitational acceleration [15].

#### 2.4.2. Gait Speed

Physical performance was assessed using the gait speed. For gait speed measurement, the participants walked barefoot at their self-selected speed on force plates until at least three trials were performed and subsequently recorded. The gait speed result was recorded in m/s using an eight-camera motion analysis system, and the results were determined using a non-dimensionalized gait speed equation:

(2) $$v\;*=v/\sqrt{gL_{leg}},$$

Where *v* is the absolute gait speed value, *L_leg_* is the length of the leg, and *g* is gravitational acceleration [15]. The mean gait speed was calculated over three trials of gait analysis for the right and left lower limbs.

### 2.5. Statistical Analyses

Power analysis for Spearman’s rank correlation analysis was conducted using G*Power (Heinrich Heine University Düsseldorf, Düsseldorf, Germany) to determine the optimal sample size for a statistical power of 0.80, alpha of 0.05, large effect size (*ρ* = 0.5), and two-tailed test. The normal distribution for each variable was confirmed using the Shapiro–Wilk test. A chi-square test was used to compare differences in proportions between the sexes in each group (GMFCS levels I and II). Data of patients with GMFCS level I and II spastic cerebral palsy were expressed as mean with standard deviations and as median values (range) and were compared using an independent *t*-test or the Mann–Whitney *U* test. Effect sizes were calculated using *r* (3,4) or Cramer’s *V*. Effect sizes with *r* > 0.1 or −0.1 were regarded as small, *r* < 0.3 or −0.3 as moderate, and *r* < 0.5 or −0.5 as large.

(3) $$\mathrm{Independent}\;t-\mathrm{test}:\;r=\sqrt{\frac{t^{2}}{t^{2}+df}},$$

(4) $$\mathrm{Mann}–\mathrm{Whitney}\;U\;\mathrm{test}:\;r=Z/\sqrt{n},$$

Sample sizes for Spearman’s correlation analyses were determined using power analysis. Spearman’s rank correlation coefficients were used to determine the associations between GDI and FTSST, GDI, and gait speed. All analyses were performed using SPSS version 23 (IBM Inc., Armonk, NY, USA). Statistical significance was set at *p* < 0.05.

## 3. Results

All participants were able to perform all evaluations irrespective of the severity of their disease. Their clinicodemographic characteristics are summarized in Table 1. No significant differences in the proportions of boys and girls were observed between the GMFCS level I and II groups (*p* = 0.89). Children with GMFCS level II spastic cerebral palsy demonstrated lower GDI (*p* < 0.001) and poorer FTSST (*p* = 0.031) results than those with GMFCS level I spastic cerebral palsy (Table 2). There were no significant differences in gait speed, age, height, weight, and body mass index (BMI) between the GMFCS level I and II groups (Table 1 and Table 2). The effect size for GDI was *r* = 0.6. The effect sizes for other variables were as follows: FTSST, *r* = −0.4; gait speed, *r* = −0.1; age, *r* = −0.3; height, *r* = 0.2; weight, *r* = −0.2; BMI, *r* = −0.2; and sex, Cramer’s *V* = 0.03. This suggests no effect between the two individual variables. FTSST was significantly negatively correlated with GDI (*r* = −0.624; *p* < 0.001; Table 3). Additionally, no significant association between GDI and gait speed findings was found.

## 4. Discussion

This study evaluated the association between GDI and FTSST results in pediatric patients with GMFCS level I and II spastic cerebral palsy. We found that FTSST results may be associated with gait impairment in these patients. Hence, FTSST could be useful in the assessment of gait impairment in pediatric patients with GMFCS level I and II spastic cerebral palsy. Furthermore, management of gait impairment is important in these patients because gait quality is associated with muscle strength of the lower leg.

Although there was a significant GMFCS level-based difference in GDI and FTSST results, our data indicated GDI-associated decreases in FTSST values. As a result, GDI scores decreased with increasing FTSST time. Accordingly, GDI may be more closely associated with lower leg muscle strength, as demonstrated by FTSST. Therefore, the difference in the performance levels of patients with GMFCS level I and II spastic cerebral palsy in FTSST may increase with a decrease in GDI. Furthermore, we found a negative correlation between GDI and FTSST values in our patients but observed no differences between gait speed in the GMFCS level I and II groups and no association between GDI and gait speed. Therefore, only a part of our hypothesis—that regarding FTSST—was confirmed. The results suggest that physical function assessment is of limited clinical value when evaluated in conjunction with GDI. However, this information should be considered during clinical decision-making because FTSST might be useful in the assessment of the effect of decreased gait quality on lower leg muscle weakness.

In the current study, motor function was assessed using FTSST, which is a validated clinical tool for assessing muscle strength in patients with GMFCS level I and II spastic cerebral palsy [9,10]. Recently, functional muscle-strength assessments of the lower leg, such as FTSST, have been proven to be beneficial in patients with spastic cerebral palsy and an essential component of clinical evaluation. Therefore, it is important to quantitatively measure the motor function in this population [9,10]. Furthermore, muscle strength was reported to be a major factor that influences gait [16,17,18]. Wang et al., reported that in patients with spastic cerebral palsy, FTSST findings correlated not only with the strengths of the primary lower extremity extensors, but also with the strengths of the trunk extensors, hip flexors, hip abductors, knee flexors, and ankle dorsiflexors [9]. These results suggest that gait quality in patients with spastic cerebral palsy who can ambulate without an assistive device is associated with FTSST, which should be evaluated in conjunction with GDI. 

Lower leg muscle strength has been demonstrated to moderately correlate with gait ability, among others, in children with cerebral palsy [19,20]. This suggests that lower leg muscle strength [16,20,21,22,23,24] and balance are important prerequisites of gait function or overall gait quality. Moreover, other studies have shown that lower leg muscle strength may play a critical role in gait [25,26]. In other words, functional decline—represented by the FTSST and GDI findings—may be prevented if gait impairment is ameliorated through exercise therapy and orthopedic treatments to address the aspects of body structure and function in pediatric patients [27,28,29,30], such as strength, balance, or bony abnormalities, particularly in those with GMFCS level I and II spastic cerebral palsy. However, gait quality and physical function in children with cerebral palsy are complex topics that warrant further investigation. Further longitudinal and interventional studies should be conducted to investigate the possibility of causality between GDI and FTSST.

The present study has some limitations that should be considered while interpreting the data. Firstly, this was a cross-sectional study; therefore, it must be emphasized that clear causal associations cannot be established based on our results. Secondly, we included participants who had undergone lower extremity orthopedic surgery more than 1 year earlier; therefore, preoperative correlation analyses of the GDI, FTSST, and gait speed findings in them were not possible. Thirdly, the assessment of muscle strength was limited to FTSST, and individual muscle strength evaluation of the lower leg muscles was not conducted. Finally, we investigated only FTSST and gait speed to evaluate motor function, and did not investigate other motor functions that could potentially contribute to gait quality. However, to the best of our knowledge, this is also the first study to investigate the association between GDI and FTSST results in pediatric patients with GMFCS level I and II spastic cerebral palsy. For better evaluation of gait quality, future longitudinal studies assessing FTSST, gait speed, and GDI findings are required. 

## 5. Conclusions 

Motor function may be important for the maintenance of gait quality in patients with GMFCS level I and II spastic cerebral palsy and should not be ignored. Therefore, FTSST results may be associated with gait ability in children with spastic cerebral palsy who can ambulate without an assistive device.

## Figures and Tables

**Table 1:** Demographic characteristics of the 35 participants.

Variable	GMFCS Level I (*n* = 14)	GMFCS Level II (*n* = 21)	*p*-Value
Age (years)	10.5 (8–16)	9.0 (5–16)	0.090
Height (cm)	134.2 ± 12.4	128.6 ± 18.6	0.328
Weight (kg)	30.2 (18.1–48.3)	24.0 (16.8–68.4)	0.337
Body mass index (kg/m^2^)	15.8 (13.7–19.9)	16.8 (13.7–26.2)	0.252
Sex: female/male	7/7	10/11	0.890

Age and weight *p*-values were derived using the Mann–Whitney *U* test. Other *p*-values were derived using an independent *t*-test. Differences in the proportions of sexes was derived using the chi-square test. Data are presented as mean ± standard deviation or as the median (range). GMFCS: Gross Motor Function Classification System.

**Table 2:** Demographic functional outcomes of the 35 participants.

Variable	GMFCS Level I (*n* = 14)	GMFCS Level II (*n* = 21)	*p*-Value
Gait Deviation Index (point)	82.2 ± 12.2	67.1 ± 11.6	0.001
Five-times-sit-to-stand test	26.5 (17.2–50.2)	41.3 (16.2–81.8)	0.031
Gait speed	0.40 (0.30–0.49)	0.40 (0.08–0.52)	0.632

The five-times-sit-to-stand test *p*-value was derived using the Mann–Whitney *U* test. Gait Deviation Index and gait speed *p*-values were derived using an independent *t*-test. Data are presented as mean ± standard deviation or as the median (range). GMFCS: Gross Motor Function Classification System.

**Table 3:** Correlation between GDI, FTSST, and gait speed according to GMFCS levels I and II (*n* = 35).

Variables	GDI	*p*-Value
FTSST	−0.624	0.001
Gait speed	−0.068	0.702

Data were generated using Spearman’s rank correlation (GDI and FTSST) coefficient analyses. FTSST, five-times-sit-to-stand test; GDI, Gait Deviation Index; GMFCS, Gross Motor Function Classification System. No significant association between FTSST and gait speed was found (*r* = −0.182; *p* < 0.304).
