# Correction: Association between Gait Deviation Index and Physical Function in Children with Bilateral Spastic Cerebral Palsy: A Cross-Sectional Study. *J. Clin. Med*. 2020, *9*, 28

**DOI:** 10.3390/jcm9020569

**Published:** 2020-02-19

**Authors:** Tadashi Ito, Koji Noritake, Hiroshi Sugiura, Yasunari Kamiya, Hidehito Tomita, Yuji Ito, Hideshi Sugiura, Nobuhiko Ochi, Yuji Yoshihashi

**Affiliations:** 1 Three-Dimensional Motion Analysis Room, Aichi Prefectural Mikawa Aoitori Medical and Rehabilitation Center for Developmental Disabilities, Okazaki 444-0002, Japan; 2 Department of Physical Therapy, Graduate School of Medicine, Nagoya University, Nagoya 461-8673, Japan; hsugiura@met.nagoya-u.ac.jp; 3 Department of Orthopedic Surgery, Aichi Prefectural Mikawa Aoitori Medical and Rehabilitation Center for Developmental Disabilities, Okazaki 444-0002, Japan; noritake@mikawa-aoitori.jp (K.N.); cedar164@ybb.ne.jp (H.S.); 4 Department of Orthopedic Surgery, Nagoya University Hospital, Nagoya 466-8560, Japan; yasunari.kamiya@gmail.com; 5 Graduate School of Health Sciences, Toyohashi Sozo University, Toyohashi 440-8511, Japan; h_tomita@sozo.ac.jp; 6 Department of Rehabilitation, Aichi Prefectural Mikawa Aoitori Medical and Rehabilitation Center for Developmental Disabilities, Okazaki 444-0002, Japan; yosihasi@mikawa-aoitori.jp; 7 Department of Pediatrics, Aichi Prefectural Mikawa Aoitori Medical and Rehabilitation Center for Developmental Disabilities, Okazaki 444-0002, Japan; yuji.ito@med.nagoya-u.ac.jp (Y.I.); aoi2pochi@yahoo.co.jp (N.O.)

The authors wish to make the following corrections to this paper [1].

(1) 2.5. Statistical Analyses

Effect sizes with *r* > 0.1 or −0.1 were regarded as small, *r* < 0.3 or −0.3 as moderate, and *r* < 0.5 or −0.5 as large.

should be replaced with

Effect sizes with *r* = 0.1 or −0.1 were regarded as small, *r* = 0.3 or −0.3 as moderate, and *r* = 0.5 or −0.5 as large.

(2) Table 3. Correlation between GDI, FTSST, and gait speed according to GMFCS levels I and II (*n* = 35).

Data were generated using Spearman’s rank correlation (GDI and FTSST) coefficient analyses. FTSST, five-times-sit-to-stand test; GDI, Gait Deviation Index; GMFCS, Gross Motor Function Classification System. No significant association between FTSST and gait speed was found (*r* = −0.182; *p* < 0.304).

should be replaced with

Data were generated using Spearman’s rank correlation (GDI and FTSST) coefficient analyses. FTSST, five-times-sit-to-stand test; GDI, Gait Deviation Index; GMFCS, Gross Motor Function Classification System. No significant association between FTSST and gait speed was found (*r* = −0.182; *p* = 0.304).

The incorrect presentation of inequality sign does not affect the results presented in the paper. The authors apologize to the readers for any inconvenience caused by these changes. It is important to state that this correction does not affect our study’s results and involve no changes or modifications in the original data supporting our results. The original manuscript will remain online on the article webpage, with reference to this Correction.
